# Supplementary material for: Detecting papilloedema as a marker of raised intracranial pressure using artificial intelligence: A systematic review
Source: PLOS Digit Health. 2025 Sep 2;4(9):e0000783. doi: 10.1371/journal.pdig.0000783 (PMC12404415; doi:10.1371/journal.pdig.0000783)
Supplement: S1 Appendix — (DOCX) [file pdig.0000783.s001.docx]

### S1 Appendix: Search strategy and definitions

| **Ovid MEDLINE Search Strategy** | |
| --- | --- |
| Terms pertaining to artificial intelligence or their subsets (machine learning, deep learning) | 1. exp Artificial Intelligence/ 2. algorithm*.mp 3. deep learning.mp 4. machine learning.mp 5. artificial intelligen*.mp 6. exp Algorithms/ |
| Terms pertaining to papilledema or other associated descriptors | 1. exp Papilledema/ 2. optic nerve swelling.mp 3. optic disc swelling.mp 4. papilledem*.mp |
| Boolean operators for search term combination | 1. 1 OR 2 OR 3 OR 4 OR 5 OR 6 2. 7 OR 8 OR 9 OR 10 3. 11 AND 12 |

| **Definitions of terms used** | |
| --- | --- |
| Artificial Intelligence | Artificial intelligence (AI) is the concept of a computer or system imitating human-like cognition, such as reasoning, problem-solving or learning. |
| Machine Learning | Machine learning (ML) is a subset of artificial intelligence, in which algorithms are created to analyse datasets and detect patterns, which can then be applied to unseen data. There are different types of ML such as supervised, unsupervised, semi-supervised, and reinforcement learning. |
| Supervised Machine Learning | Supervised ML uses labelled datasets and learns to patterns or identifies relationships by forming input-output pairs. Decision trees, random forest classifiers, and support vector machine algorithms are few examples of types of supervised ML. |
| Deep Learning Systems | Deep learning systems (DLS) refer to a subset of machine learning that uses artificial neural networks (ANNs). ANNs are based on the structure of biological brains and are composed of multiple interconnected ‘nodes’ (or neurons), connected by ‘edges’ (or synapses). Multiple hidden, or intermediate, layers perform different functions and connect the input layer to the output layer, A neural network is defined as ‘deep’ if it has two or more hidden layers. It can be unsupervised, supervised, or have components of both. |
| Convolutional Neural Networks | Convolutional neural network (CNN) is a type of DLS. CNNs use different kernels or filters that can be applied to detect different features. Multiple convolution layers allow the system to learn more complex features from the input. Non-linear activation functions are applied between layers. The pooling layer compresses the output of the convolution and enables the detection of larger-scale features. The fully connected layer then applies weights to all of the convolutions in order to determine the probability of each output. |
