# Supplementary material for: Detecting papilloedema as a marker of raised intracranial pressure using artificial intelligence: A systematic review
Source: PLOS Digit Health. 2025 Sep 2;4(9):e0000783. doi: 10.1371/journal.pdig.0000783 (PMC12404415; doi:10.1371/journal.pdig.0000783)

### S3 Appendix: Assessment of Reporting Quality using Checklist for Artificial Intelligence in Medical Imaging

*Reporting of title and abstract*

The consistency of reporting quality in title and abstract was varied, with only half of the studies meeting the checklist’s criteria (figure 2). Common factors contributing to poor quality of reporting included: 1) lack of a standardised structure for reporting, 2) failure to provide key details regarding the demographics of the study population or study design, 3) omission of significance (p) values and/or confidence interval for comparisons, and 4) lack of disclosure on the public availability of software, data or model code.

*Reporting of introduction*

The majority of studies outlined their rationale, objectives, and anticipated impact (see figure 2). However, a subset of studies (n=8) provided limited reference to existing literature in the field, despite being conducted more recently, and lacked clarity on how their study was different from previous research.

*Reporting of methodology*

CLAIM recommends that study methodology should be described ‘with sufficient detail for readers to reproduce the study and provide full details in a supplement if description exceeds journal’s word limit’. However, only a small fraction of studies adhered to such a rigorous standard of reporting, and none used datasets that are publicly accessible for testing the reproducibility of their findings ^1,2^.

Specific areas that were reported well by a majority of the studies included details on the source of the data, pre-processing steps, model description, data partitioning, and methods used for selecting the best performing model if more than one model was utilised. However, it was uncommon for authors to provide ‘the structure of the model in code’ which would help define the functions, layers and connections to reconstruct the network. Less well reported areas included information on the eligibility criteria, handling of missing data, clear rationale for and definition of ground truth, and the use of any power calculations for determining sample size. Methods for labelling of images, anonymisation of data, and reduction of inter- and intra- rater variability, were also poorly reported. Information on any software libraries, frameworks, and packages used were reported in detail by ten studies and partially reported by six. Metrics used for measuring performance were reported by all studies, although seven studies did not provide uncertainty estimates. Seven studies described at least one method for explainability or interpretability such as by the use of saliency maps.

*Reporting of results*

Clinical and demographic data were missing from most of the studies, with only seven studies providing information on both age and gender as a minimum. Ethnicity data was formally collected by only two studies, though it was not available for all images included in the study and the split between the different ethnicities was not reported (https://pmc.ncbi.nlm.nih.gov/articles/PMC11046195/) ^1^. Two studies partially report the ethnic background of their study cohort^1,3^.Though model performance was reported by all the studies, benchmarking the performance of the AI model against current standards such as by comparing its performance with medical experts was rarely performed. Analysis of incorrectly classified cases was performed only by eleven studies.

*Reporting of discussion*

Nearly all the studies (n=21) included in the paper discussed the limitations of their model. However, comparison of their model’s performance with existing models was limited and a discussion on statistical uncertainty, generalisability, and implications for practice was limited.

*Reporting of other information*

Four studies provided additional information in the form of either publicly available algorithm code on GitHub or detailed supplementary files with sufficient information that readers can evaluate the validity of the study. Very few studies (n=4) specified the source of funding and the funders’ role in performing the study.

**Mean quality of reporting by CLAIM item across all studies**


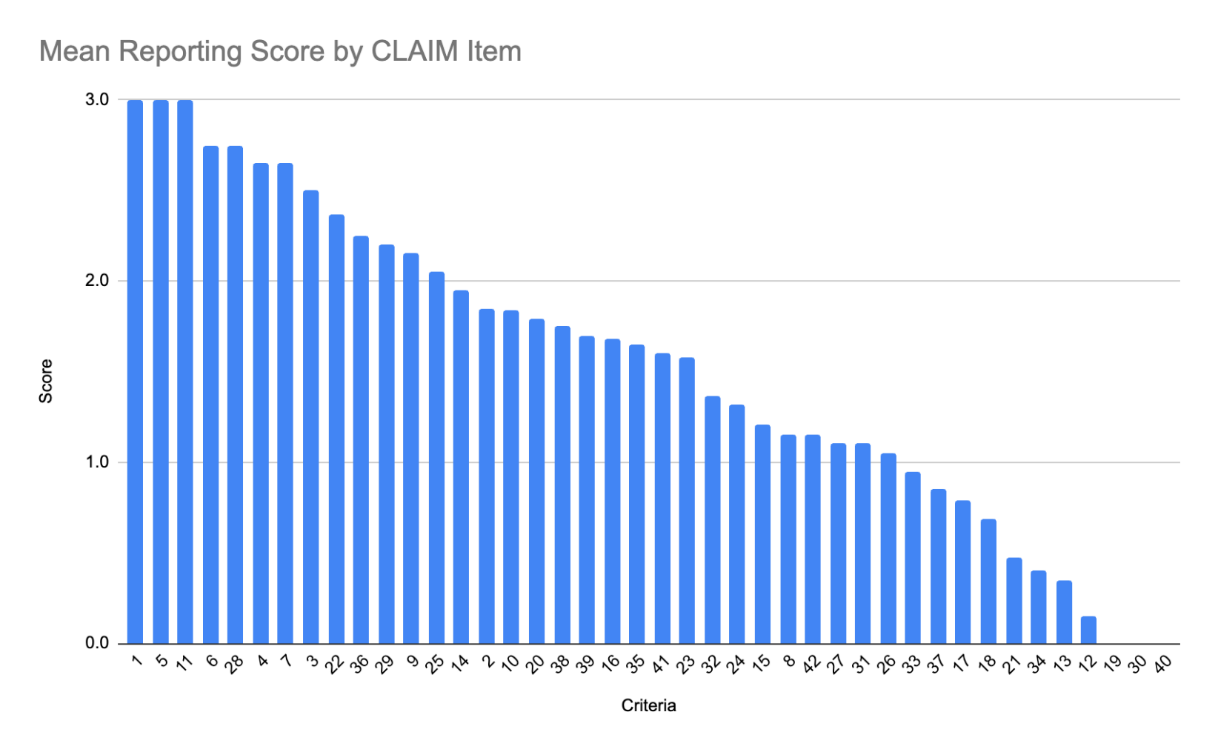


The item number on the x-axis corresponds to the item number on the Checklist for Artificial Intelligence in Medical Imaging (CLAIM) produced by Mongan et al^4^. A copy of the checklist can be found below:


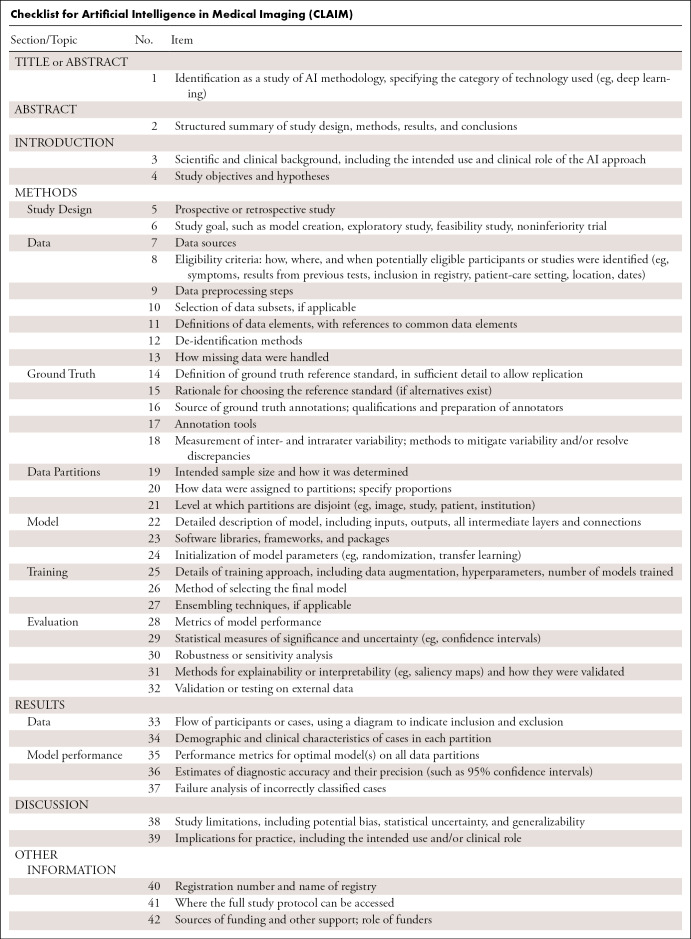

Supplement: S3 Appendix — (DOCX) [file pdig.0000783.s003.docx]
