# Supplementary material for: Detecting papilloedema as a marker of raised intracranial pressure using artificial intelligence: A systematic review
Source: PLOS Digit Health. 2025 Sep 2;4(9):e0000783. doi: 10.1371/journal.pdig.0000783 (PMC12404415; doi:10.1371/journal.pdig.0000783)
Supplement: S4 Appendix — (DOCX) [file pdig.0000783.s004.docx]

**S4 Appendix: Commonly used metrics for measuring performance and their definition.**

Definitions for accuracy, AUROC, precision and recall were adapted from Google’s Machine Learning Crash Course.

| **Performance metric** | **Definition** | **Formulae** |
| --- | --- | --- |
| **Accuracy** | Ratio of number of correct predictions to the total number of predictions made. | TP (True positive) + TN (true negative) / TP (true positive) +TN (true negative) + FP (false positive) +FN (false negative) |
| **Area Under Receiving Operator Characteristics’ Curve** | AUC measures the area underneath the Receiving Operator Characteristics Curve, which is a graph that plots true positive rate (TPR), also known as sensitivity, against a false positive rate (FPR) which is 1 - specificity at different classification thresholds. | TPR or Sensitivity = TP/FN + TP  FPR or 1 - Specificity = FP/TN+FP  Area measured through integral |
| **Sensitivity or Recall** | Measures model’s ability to measure a true positive | As above |
| **Specificity** | Measures model’s ability to predict a true negative. | TN/TN+FP |
| **Precision** | Aims to identify what proportion of positive predictions made by the model was actually correctly identified. | TP/TP+FP |
| **F1 score or Dice score** | Harmonic mean of precision and recall. | 2 x Precision x Recall / Precision + Recall = 2TP / 2TP + FP + FN |
| **Positive Predictive Value** | Aims to answer how likely a positive test is actually a true positive | TP / TP + FP |
| **Negative Predictive Value** | Aims to answer how likely a negative test is actually a true negative | TN / TN + FN |
| **Cohen’s weighted K coefficient** | Measures agreement between two raters^5^. | See reference 66. |
