# Supplementary material for: Detecting papilloedema as a marker of raised intracranial pressure using artificial intelligence: A systematic review
Source: PLOS Digit Health. 2025 Sep 2;4(9):e0000783. doi: 10.1371/journal.pdig.0000783 (PMC12404415; doi:10.1371/journal.pdig.0000783)
Supplement: S5 Appendix — (DOCX) [file pdig.0000783.s005.docx]

**S5 Appendix: Supplementary data tables**

**Table A: Summary of number of normal retinal and papilloedema images in the deep learning systems**

| **Author, year** | **Number of normal retinal images in testing and training** | **Number of images of papilloedema in training and internal testing** | **Number of normal retinal images for external testing if performed** | **Number of images of papilloedema for external testing if performed** |
| --- | --- | --- | --- | --- |
| Ahn et al., 2019 | Training: 505  Validation: 155  Testing: 119 | Training: 174  Validation: 66  Testing: 55 | Not performed. | Not performed |
| Biousse et al., 2020 | Training: 9,156 Testing: 400 | Training: 2,148 Testing: 201 | Not performed | Not performed |
| Quellec et al., 2020 | Training: 3992  Validation: 2890  Testing: 10,073 | Training: 0  Validation: 20  Testing: 79 | Unclear if testing data was an external dataset. | Unclear if testing data was an external dataset. |
| Milea et al., 2020 | Training: 9156  Testing: 613 | Training: 2148  Testing: 360 | 613 | 360 |
| Cao et al., 2021 | 1032 images included in total. Split of normal retina compared papilloedema images not reported. | | | |
| Liu et al., 2021 | Training: 580 | Training: ~83 | 80 | ~23 |
| Saba et al., 2021 | Testing: 60 | Testing: 40 | Not performed | Not performed |
| Vasseneix et al., 2021 | 0 | Training: 2103 | 0 | 214 |
| Avramidis et al., 2022 | 0  (Pseudopapilloedema images used = 182) | 149 | Not performed | Not performed |
| Li et al., 2022 | Training: 46501  Validation: 10237  Internal test set: 1053 | Training: 2882  Validation: 682  Internal test set: 228 | External test set A: 441  External test set B:  1804 | External test set A: 78  External test set B: 82 |
| Sathianvichitr et al. 2022 | N/A | 3230 | N/A | 214 |
| Thiagarajan and Suguna, 2023 | Between 750-800 (only a graph provided) | ~300 | Not performed | Not performed |
| Chan et al., 2023 | Training: 2509 | Training: 480 | 579 | 57 |
| Vasseneix et al., 2023 | 400 | 201 | Not performed | Not performed |
| Salaheldin et al., 2023 | 12782 fundus images including normal retina, papilloedema, and pseudopailloedema | | 875 | 1139 |
| Biousse et al., 2024 | N/A | N/A | 1378 | 50 |
| Chang et al., 2024 | N/A | 304 | N/A | 76 |
| Branco et al., 2024 | N/A | 5908 | N/A | 2979 |
| Lin et al., 2024 | N/A | N/A | 558 | 254 |

**Table B: Summary of datasets used by non-DLS ML models**

| **Author, year** | **Number of normal retinal images in testing and training** | **Number of images of papilloedema in training and internal testing** | **External validation** | **Number of datasets used for evaluating performance** |
| --- | --- | --- | --- | --- |
| Echegaray et al., 2011 | 10 | 86 | Not performed | 1 |
| Akbar et al., Mar 2017 | 90 | 70 | Not performed | 2 |
| Akbar et al., Nov 2017 | Not reported | Not reported | Not performed | 2 |
| Fatima et al. 2017 | 90 | 70 | Not performed | 2 |

**Table C: Summary of key reported metrics of performance**

| **Author, year** | **Model type** | **Outcome of interest** | **AUROC** | **Accuracy** | **Sensitivity** | **Specificity** |
| --- | --- | --- | --- | --- | --- | --- |
| Ahn et al., 2019 | DLS | Distinguishing pseudopapilloedema from a mixed dataset containing papilloedema images and other optic neuropathies | Training dataset: 1.0 Validation dataset: 0.98  Testing dataset: 0.99 | Training dataset: 100% Validation dataset: 96.3%  Testing dataset: 95.8% | NR | NR |
| Biousse et al., 2020 | DLS | Papilloedema detection in a dataset containing normal retina and other optic disc abnormalities; performance compared with 2 expert neuro-ophthalmologists | NR | 91.5% (95% CI 89.6-93.4) | 83.1% (95% CI 77.9-88.3) | 94.3% (95% CI 92.5-96.2) |
| Milea et al., 2020 | DLS | Papilloedema detection in a mixed dataset containing normal retina and other optic disc abnormalities | Validation dataset: 0.99 (95% CI 0.98-0.99) External dataset: 0.96 (95% CI 0.95-0.97) | Validation dataset: 94.8% (95% CI 94.4-95.3) External dataset: 87.5% (95% CI 85.5-89.3) | Validation dataset: 93.2% (95% CI 91.8% - 94.5) External dataset: 96.4% (95% CI 93.9-98.3) | Validation dataset: 95.1% (95% CI 94.7-95.6) External dataset: 84.7% (95% CI 82.3-87.1) |
| Quellec et al., 2020 | DLS | Papilloedema detection in a mixed dataset containing normal retina and 41 different ophthalmological conditions | 0.95 | NR | NR | NR |
| Cao et al., 2021 | DLS | Papilloedema detection in a mixed dataset containing normal retinal images 4 other optic neuropathies | NR | 88.6% | 86.4% (Reported as recall) | NR |
| Saba et al., 2021 | DLS | Papilloedema detection in a dataset containing normal retinal images and papilloedema | NR | 99.1% | 98.6% | 97.8% |
| Vasseneix et al., 2021 | DLS | Distinguishing severity of severe papilloedema from mild to moderate papilloedema | 0.93 (95% CI 0.89-0.96) | 87.9% (95% CI 82.7-91.9) | 91.8% (95% CI 86.9-96.7) | 82.6% (95% CI 74.9-90.4) |
| Avramidis et al., 2022 | DLS | Papilloedema detection in a dataset containing papilloedema and pseudopapilloedema images | 0.79 +/- 0.007 | 0.8 +/- 0.008 | NR | NR |
| Li B et al., 2022 | DLS | Papilloedema detection in a mixed dataset containing normal retina and 12 other ophthalmological conditions | Internal test set: 0.98 (95% CI 0.975 - 0.985)External test set A: 0.991 (95% CI 0.989 - 0.994) External test set B: 0.99 (95% CI 0.988 - 0.992) | NR | Internal test set: 0.904 (95% CI 0.943-0.958)External test set A: 0.756 (95% CI 0.742-0.77) External test set B: 0.756 (95% CI 0.748-0.764) | Internal test set: 0.95 (95% CI 0.943 - 0.957)  External test set A: 0.99 (95% CI 0.986 - 0.993)  External test set B: 0.975 (95% CI 0.972-0.978) |
| Chan et al., 2023 | DLS | Classification of quality of images in a mixed dataset containing normal retinal images, papilloedema images, and other optic disc abnormalities | Good: 0.93 Borderline: 0.9 Poor: 1.00 | Good: 93.8% Borderline: 90.6%  Poor: 99.1% | Good: 90.6% Borderline: 65.4% Poor: 81.5% | Good: 75.9% Borderline: 93.4%  Poor: 99.7% |
| Thiagarajan and Suguna, 2023 | DLS | Papilloedema detection in a mixed dataset containing normal retina and pseudopapilloedema images | NR | 0.98 | NR | NR |
| Biousse et al., 2024 | DLS | Detection of papilloedema in nonmydriatic fundus photographs | 0.97 | 76.6% | 84% | 98.9% |
| Branco et al., 2024 | DLS | Assignment of a Frisén grade to fundus photos of papilloedema | NR | NR | NR | NR |
| Chang et al., 2024 | DLS | Differentiating paediatric pseudopapilloedema from true papilloedema in fundus photographs | 0.77 | 70% | 73.4% | 67.3% |
| Lin et al., 2024 | DLS | Identifying papilloedema and other optic disc abnormalities in paediatric fundus photographs | 0.99 | 89.6% | 98% | 94.1% |
| Salaheldin et al., 2024 | DLS | Identification of papilloedema from fundus photographs | NR | 95.45% for multi paths94.81 for LSTM | 93.16 for multi paths92.19 for LSTM | 96.62 for multi paths96.12 for LSTM |
| Sathianvichitr et al., 2024 | DLS | Binary classification of optic disc drusen and papilloedema on mydriatic fundus photographs | 0.99 (95% CI 0.98-1.00) for validation0.98 (95%CI 0.97-0.99) for internal testing0.97 (0.96-0.98) for external validation | 93.2 (91.1-94.7)External Validation - 90.5% (88-92.9) | 91.9% (CI 90.1-94.3)External Validation - 86% (82.1-90.1) | 93.9 (90.1-97.3)External Validation - 94.9 (92.3-97.6) |
| Echegaray et al., 2011 | Supervised ML with decision tree forest | Papilloedema detection and grading in a dataset with normal retinal images | NR | NR | NR | NR |
| Fatima et al., 2017 | Supervised ML with SVM | Papilloedema detection in a dataset with normal retina images | NR | 85.8% | 83.9% | 88.3% |
| Akbar et al., 2017 March | Supervised ML with SVM and RBF | Papilloedema detection in a dataset with normal retina images | NR | 92.8 +/- 1.1% | 90 +/- 2.3% | 96.4 +/- 1.9% |
| Akbar et al., 2017 November | Supervised ML with SVM and RBF | Papilloedema detection in a mixed dataset containing normal retina and retinal images with features of hypertensive retinopathy | NR | STARE dataset: 95.9%Local AVRDB dataset: 97.5% | STARE dataset: 96.7%Local AVRDB dataset: 98.9% | STARE dataset: 95.9%Local AVRDB dataset: 97.3% |
| Johnson et al., 2018 | Supervised ML with random forest bagged ensemble regression model | Estimation of degree of papilloedema from retinal images | NR | NR | NR | NR |
| Keerthiveena et al., 2019 | Supervised ML with SVM | Detection of multiple diseases including papilloedema from mixed datasets with 4 other conditions | 0.97 | 96.3% | 95.8% | 97.8% |
| Naing and Aimmanee, 2024 | Supervised ML with SVM | Optic disc oedema classification | NR | 99.4% | NR | NR |
| Hagen et al., 2023 | Linear mixed model with a DLS element | Estimating CSF pressure using retinal vessel dynamic features of papilloedema | 0.81* | NR | 78% | 92% |

***** for a cut off value of <0.71 as the arteriole-to-venule- diameter for an increased lumbar opening pressure of > 20mm Hg in patients with suspected idiopathic intracranial hypertension
