# Supplementary material for: Detecting papilloedema as a marker of raised intracranial pressure using artificial intelligence: A systematic review
Source: PLOS Digit Health. 2025 Sep 2;4(9):e0000783. doi: 10.1371/journal.pdig.0000783 (PMC12404415; doi:10.1371/journal.pdig.0000783)
Supplement: S6 Appendix — (DOCX) [file pdig.0000783.s006.docx]

**S6 Appendix: Table of articles screened and reasons for exclusion**

The data extractors were LR, BGS, and TE. LR and BGS extracted data on 21st April 2023, whereas BGS and TE extracted data on 11^th^ October 2024. Please click this link to access the table of articles screened during our search and reasons for exclusion: <https://docs.google.com/spreadsheets/d/1kKqJz4BC_qKcbUBOmcfNrR32Jd-drt_A/edit?usp=drive_link&ouid=113729853216054956057&rtpof=true&sd=true>

**REFERENCES:**

1 Milea, D. *et al.* Artificial intelligence to detect papilledema from ocular fundus photographs. *New Engl. J. Med.* **382**, 1687-1695, doi:10.1056/NEJMoa1917130 (2020).

2 Quellec, G., Lamard, M., Conze, P.-H., Massin, P. & Cochener, B. Automatic detection of rare pathologies in fundus photographs using few-shot learning. *Med. Image Anal.* **61**, 101660, doi:<https://doi.org/10.1016/j.media.2020.101660> (2020).

3 Liu, T. Y. A. *et al.* Detection of Optic Disc Abnormalities in Color Fundus Photographs Using Deep Learning. *J Neuroophthalmol* **41**, 368-374, doi:10.1097/WNO.0000000000001358 (2021).

4 Mongan, J., Moy, L. & Charles E. Kahn, J. Checklist for Artificial Intelligence in Medical Imaging (CLAIM): A Guide for Authors and Reviewers. *Radiology: Artificial Intelligence* **2**, e200029, doi:10.1148/ryai.2020200029 (2020).

5 Ben-David, A. Comparison of classification accuracy using Cohen’s Weighted Kappa. *Expert Systems with Applications* **34**, 825-832, doi:<https://doi.org/10.1016/j.eswa.2006.10.022> (2008).
