# Supplementary material for: Detecting papilloedema as a marker of raised intracranial pressure using artificial intelligence: A systematic review
Source: PLOS Digit Health. 2025 Sep 2;4(9):e0000783. doi: 10.1371/journal.pdig.0000783 (PMC12404415; doi:10.1371/journal.pdig.0000783)
Supplement: Attachment — Submitted filename: Response to Reviewers.docx [file pdig.0000783.s008.docx]

**Review Comments to the Author**

Please use the space provided to explain your answers to the questions above. You may also include additional comments for the author, including concerns about dual publication, research ethics, or publication ethics. (Please upload your review as an attachment if it exceeds 20,000 characters)

Reviewer #1: This systematic review evaluated the use of AI for detecting papilloedema from retinal images, analyzing 27 systems (19 deep learning, 8 non-deep learning). Studies showed high variability in training/testing image numbers, with limited demographic data and only 10 studies performing external validation. Pooled sensitivity and specificity were 0.87 and 0.90, respectively. Despite promising performance, data bias, poor reporting, and lack of reproducibility limit confidence. AI models could support papilloedema triage in resource-limited settings, but robust external validation across diverse populations and clinical environments is essential before routine clinical adoption can be recommended. This review is interesting and valuable, however there is a few concerns to publish in current version.

We really appreciate the reviewer’s opinion that this is a valuable piece of work, and have accommodated their very helpful suggestions to improve the manuscript.

Major concerns
 1. The authors correctly highlight that many studies fail to meet the CLAIM criteria. However, the underlying issue is that a significant proportion of studies were not originally designed to conform to these criteria. In other words, the authors are raising the important question of whether CLAIM itself represents the optimal standard for evaluating these studies. I agree that the approach of seeking the most appropriate evaluation framework, rather than rigidly adhering to one specific set of criteria, is appropriate.

Thank you for your comment raising the importance of whether CLAIM is an optimal standard for evaluating the studies. While alternative frameworks such as CONSORT-AI, DECIDE-AI, STARD-AI, SPIRIT-AI, and TRIPOD-AI are available, CLAIM was selected as it is specifically tailored for assessing reporting quality in medical image analysis studies that use AI. We share your concerns about the current lack of a universally accepted reporting standard for AI studies, which reflects the heterogeneity in study design of AI studies. We have explained our rationale for choosing CLAIM in our revised methodology section, and try to show that this was chosen retrospectively as the most useful, but by no means ideal, standard against which to judge papers.

2. The authors also emphasize the importance of generating high-quality fundus images without the need for pupil dilation or specialist optometry, ensuring the safe transmission and storage of these images in large, collatable datasets, and using expert annotations to assess health and disease states and to develop benchmark tools. These are all critical points. However, given current practical and logistical constraints, it may be unrealistic to expect widespread adoption of these processes in the near future. It would be valuable if the authors could propose strategies for leveraging the insights gained from the substantial body of existing research and explore potential methods for integrating and harmonizing this body of work with future studies.

This is a critical point. As this is already a long and comprehensive review, we initially aimed to avoid proposing too much in the interests of space and clarity. However, given that this has been raised we have added text in the discussion highlighting the importance of making use of processing techniques to enhance model performance.

3. The authors highlight image quality as a recurring issue — a point that has been previously noted in the literature. To strengthen this argument, it would be beneficial to explicitly reference prior studies that have addressed the challenges of image standardization across different manufacturers and imaging platforms. Addressing this industry-wide inconsistency with supporting citations would help contextualize the problem and underscore the need for standardized imaging protocols.

We have sought to address this with a small text entry on pre-processing strategies, supported by a range of citations, that could be used for improving image quality under discussion. Hopefully this sufficient to direct readers to the topic area without further extensive word count.

Reviewer #2: This systematic review addresses an important clinical problem: the automated detection of papilloedema an ocular manifestation of raised intracranial pressure using artificial intelligence (AI) applied to retinal images. Given the potential impact on triage in resource‐limited settings, the review is timely and relevant. You comprehensively searched multiple databases and included both deep learning and traditional machine learning studies. You assessed the quality of reporting using established checklists (CLAIM) and introduced a novel rubric (SMART) to appraise bias and methodological robustness. Furthermore, the manuscript presents summary statistics and meta-analytic estimates of diagnostic performance. Overall, the work is ambitious in scope and offers valuable insights into the current state of AI applications in papilloedema detection.

Thanks so much for this validation of the our scope and topic area. Like the reviewer we feel the work is and have tried to hard to address their suggestions.

Below are some strengths.

Major Strengths

1. Clinical Relevance and Innovation:
 The review targets a crucial unmet need—rapid and accurate identification of raised intracranial pressure via papilloedema detection, which could be transformative in low-resource settings. The integration of AI into bedside ophthalmic screening is both innovative and of high clinical importance.

2. Methodological Rigour:
 The authors have followed systematic review standards (PRISMA 2020) and prospectively registered the protocol with PROSPERO. Their search strategy spans four major databases, ensuring a wide capture of the relevant literature.

3. Dual Assessment of Reporting Quality and Bias:
 The use of the CLAIM checklist for assessing reporting standards is commendable. Moreover, introducing the SMART rubric to identify potential biases in AI studies adds depth to the evaluation and highlights key areas needing improvement in the primary literature.

4. Quantitative Synthesis:
 The meta-analysis of sensitivity and specificity provides an aggregated measure of diagnostic performance. Reporting pooled estimates helps readers understand the overall accuracy of AI systems, even though heterogeneity remains a challenge.

5. Comprehensive Data Extraction:
 The manuscript offers detailed tables summarizing study characteristics, dataset attributes, and model performance metrics. Such granularity is useful for readers seeking to understand the landscape of methodologies and performance variations.

This is really helpful positive feedback, particularly for our future work in this area. Thank you.

Also here are some weaknesses I think should be addressed

Major Weaknesses

1. Heterogeneity and Limited External Validation:
 A recurring issue highlighted by the review is the significant heterogeneity among studies—ranging from dataset size to image quality and annotation standards. Only a fraction of studies performed external validation, which raises concerns about the generalizability of the reported performance metrics. The review would benefit from a more in-depth discussion of how heterogeneity impacts the meta-analytic estimates and the potential for publication bias.

This is a very valid point and – as with other points made earlier by Reviewer 1 - one which we discussed during the review process but omitted due to space constraints and concerns over accessibility if trying to cover too much ground. We have mentioned a further limitation in our discussion which covers how heterogeneity impacts meta-analytic estimates in AI studies in particular – this is a topic which might really benefit from accompanying Editorial alongside this review which we would be very happy to provide.

2. Quality of Reporting in Primary Studies:
 The review notes that many primary studies do not fully adhere to reporting standards, especially in the results sections where clinical and demographic data are often missing. This limits the ability to critically evaluate the validity of the AI models. The manuscript could further explore how this poor reporting may bias the meta-analysis results.

Again, we now discuss this as a limitation in our discussion section.

3. Inclusion and Exclusion Criteria:
 Although the review details the screening process, it is not entirely clear whether the inclusion criteria for studies (e.g., types of retinal images, definitions of papilloedema) were sufficiently stringent. Greater clarity regarding the PCC criteria would strengthen the review’s reproducibility.

Agreed – we have published the protocol for the paper separately here which we refer to in our manuscript as well, which provides further detail about the inclusion criteria – <https://pubmed.ncbi.nlm.nih.gov/38433865/>.

4. Novel SMART Rubric:
 While the SMART rubric is an innovative tool for assessing bias in AI studies, its development and validation are not described in sufficient detail. More information on its derivation, weighting, and inter-rater reliability would increase confidence in its use and findings.

We have included additional text in the methods section describing the SMART tool and its adaptation. We have tried to trade-off details against length of article, and hope that this strikes the right balance.

5. Discussion of Clinical Implementation:
 Although the review acknowledges that AI models show promise, there is limited discussion on the practical challenges of translating these findings into clinical practice. Topics such as regulatory approval, integration with existing ophthalmic devices, and the need for prospective validation in diverse populations deserve further exploration.

Thank you, we agree that this is an important area worthy of deeper exploration when discussing AI models. However, these topics fall outside the scope of this focussed review and would significantly expands its length – they would again make for an excellent Editorial discussion alongside this review.

Here are some Suggestions for Improvement

1. Clarify Heterogeneity and Its Impact:
 Expand the discussion on how variability in training/testing dataset sizes, image quality, and annotation methods might affect pooled sensitivity and specificity. Consider performing subgroup analyses (if data permit) to determine whether certain study characteristics correlate with better performance metrics.

The impact of heterogeneity is now discussed at greater length under discussion. After consideration, we have found that the number of studies included in the meta-analysis is unfortunately too limited to conduct any sub-group analyses that would yield meaningful conclusions.

2. Enhance Reporting on Bias Assessment:
 Provide a more detailed description of the SMART rubric, including its domains, scoring system, and validation process. Discuss how the rubric’s findings compare with those from other established bias tools in AI research.

We have included additional text in the methods section describing its adaptation, and again would be happy to develop this thinking in an accompanying Editorial exploring the risk of bias in AI studies.

3. Address External Validity:
 Emphasise the need for prospective studies with external validation. Consider adding recommendations for future research, such as multi-center collaborations and the use of standardized imaging protocols, to improve the generalizability of AI algorithms.

We would love to develop these points. Given the length of the review (which we are aware is already at the longer end and is both detailed and technical) we have tried to avoid too many recommendations other than those very directly suggested form the data. We think there is scope to develop these in an accompanying article, drawing on the findings of this work.

4. Improve the Discussion Section:
 The discussion would benefit from a deeper exploration of the clinical implications of the findings. How might the integration of these AI systems impact current diagnostic workflows? What are the potential cost-benefit and ethical considerations when implementing AI in low-resource settings?

We agree that these would be important points of discussion. In our analysis, only a small proportion of studies (n=3) discussed how their models would reduce the workload of trained specialists. No study provided a description of its integration with existing workflows or demonstrated efficacy in improving clinical outcomes. This limits our ability to assess the potential impact of integrating AI systems to current diagnostic workflow. Nonetheless, this remains an important consideration – particularly in low-resource settings, where deployment decisions must account for cost-effectiveness and ethical implications.

5. Limitations of the Meta-analysis:
 Acknowledge the limitations inherent in aggregating data from studies with diverse methodologies and reporting standards. Discuss potential biases in study selection (e.g., publication bias) and how they might affect the pooled diagnostic accuracy.

To acknowledge this limitation, we have included more detail on how variation in standards of reporting, publication bias, variation in performance metrics used by authors, and limited access to underlying datasets can all affect our ability to draw more conclusive insights from the pooled diagnostic accuracy.

6. Future Research Directions:
 Outline concrete recommendations for improving study design in future research. This might include advocating for larger, diverse datasets, clearer definitions of papilloedema, standardized reporting guidelines, and the inclusion of clinical outcomes beyond imaging metrics.

We very much agree with these points, but were concerned about making strong recommendations which went beyond the bounds of the literature we have assessed. However we would also like to see these points raised in the literature and in any accompanying opinion piece.

Minor Comments

- Terminology Consistency:
 Ensure that terms such as “papilloedema,” “papilledema,” and “optic disc swelling” are used consistently throughout the manuscript to avoid confusion.

This has been corrected throughout the manuscript to use the term ‘papilloedema’.

- Formatting and Tables:
 Some tables (e.g., those summarizing dataset characteristics and performance metrics) are very dense. Consider simplifying or summarizing key points in the text, with detailed tables provided in supplementary materials.

Thank you – we have moved tables 3, 4 and 6 to the supplementary files.

- Reference Standard Discussion:
 The manuscript could benefit from a clearer explanation of the various “ground truth” methods used across studies. A comparative analysis of these methods would help elucidate their impact on reported performance.

We have provided explanation of the various ‘ground truth’ methods used across studies under results in section 3.1.4.

- Ethnicity and Demographics:
 Given the observed under-reporting of participant demographics, particularly ethnicity, the authors should stress the importance of collecting and reporting these data to understand model performance

This has now been addressed under discussion of the manuscript

Conclusion

This systematic review represents an important contribution to the field of AI in ophthalmology, specifically for the detection of papilloedema as a marker for raised intracranial pressure. The comprehensive search strategy, dual approach to quality and bias assessment, and quantitative synthesis of diagnostic performance are notable strengths. However, the manuscript also highlights significant challenges, including heterogeneity among studies, limited external validation, and suboptimal reporting standards. Addressing these issues in future research is critical for the translation of AI models into clinical practice.

Overall, with revisions to address the concerns noted above—particularly a deeper discussion on heterogeneity, external validity, and the clinical implementation of AI systems—the manuscript has the potential to serve as a valuable resource for both clinicians and researchers working at the intersection of neuro-ophthalmology and artificial intelligence.

I hope these comments are helpful in strengthening your manuscript.

Thank you so much for these really detailed and constructive comments which we have tried hard to address without greatly lengthening the article. On reflection, we feel that the comments both you and other reviewers have raised around some of the nuances of the discussion prompted by our findings would sit really well in an accompanying piece and we hope the journal editors might encourage submission of such an Editorial; we would be very happy to either compose this, or to nominate colleagues who might be well placed to raise these key points for the PLOS Digital Health readership.

Reviewer #3: This systematic review addresses a highly relevant and clinically significant topic—papilledema detection as an importante marker for elevated intracranial pressure. Given the potentially life-threatening nature of conditions associated with papilledema, timely and accurate identification is essential for appropriate management.

The authors provide a comprehensive and thoughtful overview of the current state of artificial intelligence applications in this area. The review thoroughly covers the imaging acquisition process, characteristics of the datasets and algorithms used, as well as technical aspects such as performance metrics. Notably, the authors also acknowledge the limitations and potential sources of bias in the existing literature, which strengthens the critical rigor of the manuscript.

To enhance the accessibility and clarity of the review for readers from non-specialist backgrounds (e.g., general practitioners, emergency physicians, or AI researchers), I suggest including illustrative figures with representative examples of papilledema and normal optic discs. Visual aids would help contextualize the clinical relevance of the findings and improve comprehension, especially for audiences without ophthalmology or neurology training.

Overall, this is a valuable contribution to the field of AI in neuro-ophthalmology.

Thank you very much for this constructive and positive review. Your comments are directly in line with those of the other peer reviewers and we hope that the changes we have made in response to their suggestions do not detract from your positive impression. We have included an additional figure illustrating the differences between a normal optic disc and papilloedema as figure 1. We have also included a figure to illustrate how retinal images can be used for training and development of AI models as figure 7. We will work with the journal in terms of visual aids to assess how we can best present our findings to make them more accessible.
